# Avian haemosporidian diversity and transmission across birds and mosquitoes in Botswana

**DOI:** 10.1016/j.ijppaw.2026.101212

**Published:** 2026-02-14

**Authors:** Antoine Perrin, Casper Nyamukondiwa, Romain Pigeault, Mmabaledi Buxton, Alexander N. Kirschel, Molly Baur, Olivier Glaizot, Philippe Christe

**Affiliations:** a Department of Ecology and Evolution, University of Lausanne, 1015, Lausanne, Switzerland; b Department of Biological Sciences and Biotechnology, Botswana International University of Science and Technology, Palapye, Botswana; c Centre for Environmental Policy, Imperial College London, Silwood Park Campus, Ascot, UK; d Department of Zoology and Entomology, Rhodes University, Makhanda, South Africa; e Ecologie & Biologie des Interactions (UMR 7267), Equipe EES, Université de Poitiers, France; f Department of Biological Sciences, Botswana University of Agriculture and Natural Resources, Gaborone, Botswana; g Department of Biological Sciences, University of Cyprus, PO Box 20537, Nicosia, 1678, Cyprus; h Natural history Museum, Department of Zoology, 1005, Lausanne, Switzerland

**Keywords:** Malaria, Migratory birds, *Plasmodium* infection, Southern africa, Vectors

## Abstract

Avian malaria parasites circulate globally among birds and their dipteran vectors, yet their diversity and transmission dynamics remain poorly characterised in sub-Saharan Africa. Here, we investigated haemosporidian infections in birds and mosquitoes across Botswana, where resident and migratory bird species interact with diverse vector communities. We screened 395 birds from 23 genera and 65 species and 1425 mosquitoes from eight genera and 28 species for *Plasmodium*, *Haemoproteus* and *Leucocytozoon*. We detected a high level of parasite diversity, identifying 40 lineages in birds, 31 in mosquitoes, including one lineage shared between the avian hosts and vectors. A total of 36 lineages were newly described. The detection of the African *Plasmodium* lineage MALNI02 in both birds and multiple mosquito taxa, provides the first evidence of its potential transmission pathway between avian hosts and vectors in Africa. Several cosmopolitan lineages previously known from Europe were detected in both resident and migratory birds and in mosquitoes. This suggests that migratory birds may help to spread these haemosporidian parasites across continents, and that African mosquito taxa can transmit them. It also indicates that infections previously detected in Europe can circulate among resident bird species in Africa. These findings support the idea of cross-transmission between resident and migratory bird species and highlight the importance of southern Africa in understanding avian malaria transmission across the African-Eurasian flyway.

## Highlights

• High diversity of haemosporidian parasites detected in both birds and mosquitoes in Botswana.
• Migratory birds may introduce cosmopolitan lineages, which can infect local mosquitoes and be carried by resident bird species.
• First evidence of potential vectors for *Plasmodium* MALNI02 in multiple African mosquito taxa.
• Local *Culex* species play a key role in facilitating parasite transmission within and among migratory and resident bird species.

## Graphical abstract

**Figure:**
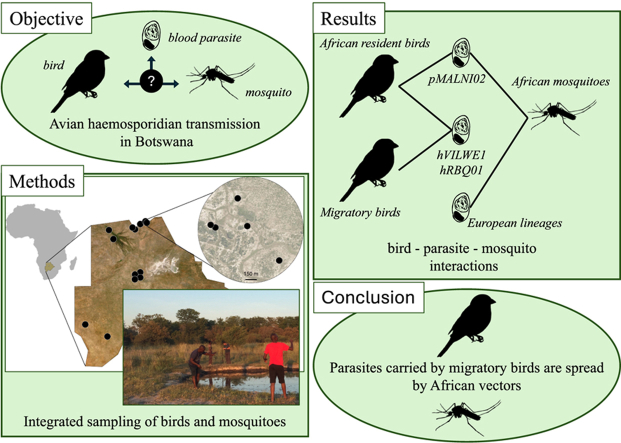

## 1 Introduction

Animal movement plays a crucial role in shaping the spatial dynamics of parasites and associated infectious diseases. Among vertebrates, migratory birds can spread their parasites along the world's major flyways (Donaldson et al., 2024; Perrin et al., 2025). Indeed, such large-scale movements can profoundly influence pathogen transmission by connecting geographically distant avian host and vector communities (Hellgren et al., 2007; Altizer et al., 2011; Dekelaita et al., 2023). Migratory birds can introduce parasites into environments where they were previously absent (Buczek et al., 2020) or alternatively experience parasite loss during migration through processes such as migratory escape, when individuals leave areas of high parasite pressure, and migratory culling, where infected individuals fail to complete migration (Bradley and Altizer, 2005; Ishtiaq and Renner, 2020). Whether migration increases or decreases parasite transmission depends on both host ecology and parasite life-history traits (Poulin and de Angeli Dutra, 2021). For vector-borne parasites, successful transmission also critically depends on the presence, abundance, and activity of competent vectors in breeding, stopover, or overwintering sites.

Avian haemosporidians of the genera *Plasmodium*, *Haemoproteus*, and *Leucocytozoon* (Apicomplexa: Haemosporida) are cosmopolitan parasites responsible for avian malaria or malaria-like infections in birds (Valkiūnas, 2005). They are transmitted mainly by dipteran vectors, mosquitoes for *Plasmodium*, biting midges for *Haemoproteus*, and blackflies for *Leucocytozoon*. As transmission requires the presence of a competent vector (Hellgren et al., 2007), the geographic distribution of vectors combined with host migratory movements gives rise to multiple possible transmission scenarios. The detection of a parasite lineage in migratory bird species alone does not allow inference of the location of transmission, as infection may have occurred at wintering or breeding areas, or at any stopover site along the migratory route. Conversely, the detection of a parasite lineage in a resident bird species provides clear evidence of local transmission, indicating the presence of a competent vector at the sampling site. When the same parasite lineage is detected in both resident and migratory bird species, this demonstrates that local transmission is occurring, while also allowing for the possibility of transmission elsewhere along the migratory route where suitable vectors occur. For instance, the widespread *Plasmodium relictum* SGS1 occurs in resident bird species in both Europe and Africa, as well as in several intercontinental migratory species (Hellgren et al., 2007). This lineage has also been repeatedly detected in *Culex pipiens* in Europe (e.g., Glaizot et al., 2012; Ferraguti et al., 2013), a mosquito species with a worldwide distribution. However, to our knowledge, *P. relictum* SGS1 has not yet been reported in *C*. *pipiens* in Africa, despite the presence of this vector in several regions of the continent (e.g., Hammami et al., 2016; Buxton et al., 2021; Makate et al., 2022). These findings highlight the importance of investigating African overwintering habitats, where transmission may occur but remains poorly documented, to gain a full picture of parasite transmission patterns.

In Europe, several haemosporidian lineages detected in bird communities do not appear to be transmitted locally because competent vector species are absent, as reported for some *Plasmodium* lineages infecting Eurasian blackcaps (Valkiūnas, 2005; Valkiūnas et al., 2015). If such lineages were to become transmissible in new regions, whether through climate-driven shifts in vector distributions or through the arrival of vectors capable of transmitting parasite lineages carried by migratory birds, they could impose substantial biological costs on naïve host communities (van Riper et al., 1986; Atkinson et al., 1995). Understanding which vectors transmit which parasite lineages, and where, is therefore crucial for anticipating potential changes in avian malaria transmission and monitoring the emergence of parasite lineages outside their current geographic ranges.

Although many studies have focused on avian hosts, vectors remain the least explored component of avian malaria systems, especially across the African continent (but see Njabo et al., 2011; Schmid et al., 2017). This implies that our understanding of mosquito ecology and their associations with haemosporidian parasites is still poorly understood in the southern region, particularly in Botswana, where these vectors have been reported (Buxton et al., 2021). However, this region encompasses a mosaic of habitats, from the swampy Okavango Delta wetlands, the semi-arid savannas and human modified habitats, that sustain a rich diversity of both birds and mosquito vectors. Recent surveys have begun to reveal a far richer culicid fauna than previously recognised, documenting diverse assemblages of *Anopheles*, *Culex*, *Aedes*, *Mansonia*, *Coquillettidia*, *Uranotaenia*, and *Mimomyia* species, including several known or suspected avian malaria vectors (Buxton et al., 2021, 2023; Makate et al., 2022). In southern Botswana, *Culex* mosquitoes dominate numerically, representing up to 88% of captures, while *Aedes aegypti* and *Anopheles* species are far less abundant (Makate et al., 2022). The Central District also harbours *Culiseta longiareolata*, a recognised natural vector of avian malaria (Buxton et al., 2023). In the Okavango Delta, Hammami et al. (2016) captured over 25,000 mosquitoes representing 32 species, with *C*. *pipiens* and *Mansonia uniformis* as dominant taxa. However, no investigation has systematically screened mosquito communities for avian haemosporidian infections in Botswana.

In this study, we investigated the diversity of haemosporidian parasites in southern Africa, focusing on both avian hosts and mosquito communities. Using data collected from a diverse bird assemblage in Botswana, including both resident and migratory species, we screened for *Plasmodium*, *Haemoproteus*, and *Leucocytozoon* lineages and characterised the sequence variation within the *cytochrome b* gene. In parallel, we surveyed local mosquito populations to detect haemosporidian infections and identify potential vectors involved in avian malaria transmission. By comparing parasite lineages detected in birds and mosquitoes and contrasting them with European records, we aimed to (i) disentangle local transmission from migratory bird-associated transmission, (ii) identify potential links between avian hosts and vectors through their parasites and infer active transmission routes, and (iii) assess the role of migratory birds in the intercontinental circulation of avian malaria parasites. This study advances our understanding of avian malaria by integrating parasite, vertebrate host, and vector data to reveal local and migratory transmission dynamics, and by clarifying how interactions between resident and migratory birds and mosquito vectors shape the intercontinental circulation of haemosporidian parasites.

## 2 Materials and methods

### 2.1 Study site, mosquito and bird collections

**Fig. 1:**
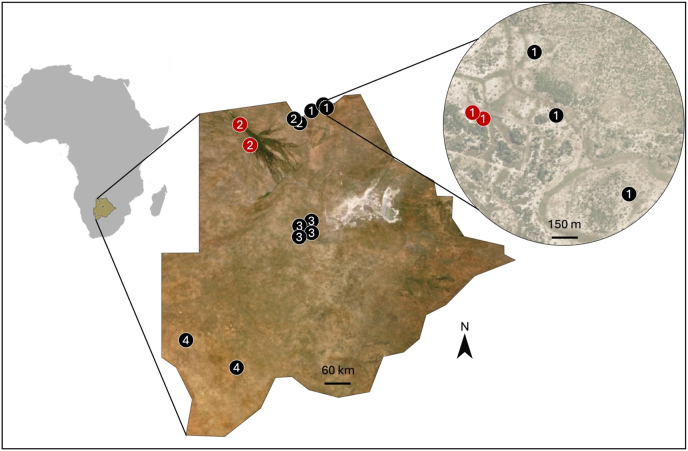
**Location of the 17 sampling sites across Botswana.** Black and red points represent sites where only mosquitoes or both birds and mosquitoes were sampled, respectively. Numbers indicate the districts: 1 = Chobe, 2 = North-West, 3 = Ghanzi, and 4 = Kgalagadi.

Field sampling took place between February and June 2019, 2020, and 2023. Mosquitoes were collected at 17 sampling sites across the Chobe, North-West, Ghanzi, and Kgalagadi Districts (Fig. 1). Birds were also captured in 2019 and 2020 at four of these sites outside National parks, in the Chobe and North-West Districts. Details of mosquito collections in 2019-2020 and site descriptions are reported in Buxton et al. (2021).

Mosquitoes were collected using six CO_2_-baited BG-Sentinel traps (Biogents AG, Regensburg, Germany) per session, set at 4pm and retrieved at 6am the following morning. Specimens were initially identified to the genus level (Jupp, 1996) and preserved in ethanol, either individually or in pools when numbers were high. Morphological and molecular identification were subsequently performed in the laboratory as described in Buxton et al. (2021). Unidentified specimens were grouped and classified to genus levels based on morphological similarity.

Birds were captured with mistnets (Ecotone, Poland) set from sunrise to 10am and from 4pm until dusk, avoiding the hottest hours of the day. Species identification followed Hancock and Weiersbye (2015) and Ryan and Sinclair (2010), with uncertain cases confirmed by *cytochrome c oxidase subunit I* (primers: LCO1490, HCO2198) and/or *cytochrome b* (primers: L14841, H15149) gene sequencing (see Supplementary Method 1 for details). Approximately 5-20 μl of blood was collected from the brachial or metacarpal vein using sterile needles and blotted onto filter paper for molecular analyses. To minimise disturbance and ensure that their natural behaviour, territorial patterns and survival were not affected, the birds were released immediately after blood collection at the site of capture.

### 2.2 DNA extraction and molecular identification of haemosporidian parasites

Total DNA was extracted from bird blood samples and mosquito thorax-abdomen pools using the DNeasy Blood & Tissue Kit (Qiagen, Hilden, Germany), following the manufacturer's protocol with two modifications for blood samples: a 45 min incubation step and final elution in 100 μL. Haemosporidian infection was detected by nested Polymerase Chain Reaction (PCR) targeting a fragment of the *cytochrome b* gene (Hellgren et al., 2004). Negative results on 2% agarose gels were confirmed with a second PCR run. PCR products were purified and sequenced as described in van Rooyen et al. (2013). Sequences were identified by local BLAST searches against the MalAvi database (Bensch et al., 2009). Mixed infections were resolved through cloning or phasing of the PCR products. Cloning followed the protocol described in van Rooyen et al. (2013). Briefly, PCR products were cloned using the pGEM-T Easy Vector System (Promega), and eight positive colonies per sample were sequenced. Only lineages detected in at least two independent colonies were retained. Phasing of mixed infections was performed manually by comparison with lineage sequences previously detected as single infections in the dataset, allowing the most parsimonious combination of co-infecting lineages to be inferred. All new lineages (≥1 bp difference from known sequences) were deposited in MalAvi.

To prevent and monitor potential contamination, all laboratory work was conducted in separate pre- and post-PCR areas. Work surfaces and equipment were regularly decontaminated using DNA removal reagents and UV irradiation. DNA extraction and PCR setup were carried out using aerosol-resistant filter tips. Negative controls were systematically included during extractions (blank) and PCR (no-template control). All negative controls consistently remained free of amplification, indicating the absence of contamination during molecular procedures.

## 3 Results

**Fig. 2:**
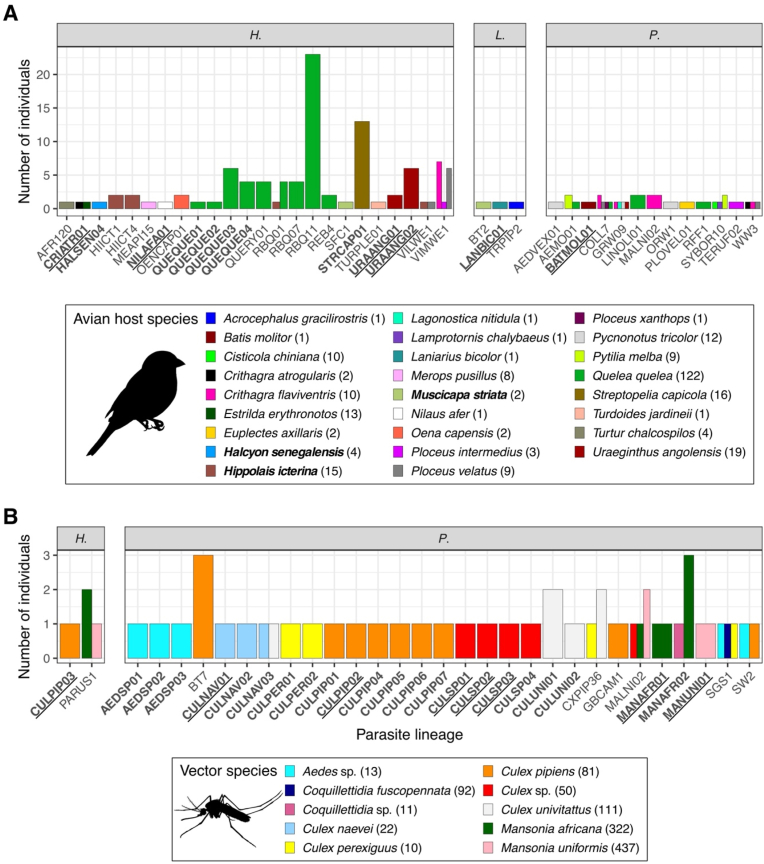
**Number of (A) birds and (B) mosquitoes infected with haemosporidian parasites (*Plasmodium* (*P.*), *Haemoproteus* (*H.*) and *Leucocytozoon* (*L.*)) per lineage.** Bars indicate the number of (A) avian host or (B) vector species associated with each lineage, with colours corresponding to individual bird or mosquito species. The number of individuals sampled for each bird or mosquito species is indicated next to species names. Parasite lineages highlighted in bold indicate newly detected lineages, and those underlined correspond to highly divergent lineages (>1% divergence) from known MalAvi sequences. Avian host species highlighted in bold correspond to Palearctic or intra-African migratory species.

A total of 395 birds from nine orders, 30 families and 65 species were captured and blood sampled. In total, 124 birds were infected by 40 distinct haemosporidian lineages, including 13 *Plasmodium*, 24 *Haemoproteus*, and three *Leucocytozoon* lineages (overall prevalence = 31%, Fig. 2A). Among these, 12 lineages were newly described, comprising 10 *Haemoproteus*, one *Plasmodium*, and one *Leucocytozoon* lineages. Six *Haemoproteus* lineages showed high similarity to previously reported haplotypes in the MalAvi database, whereas the remaining new lineages were genetically divergent (>1 % sequence divergence) from known lineages. Mixed infections were detected in 15 birds, five were resolved by cloning, while the remaining mixed infections were resolved by manual phasing. Infection prevalence for each bird species is provided in Supplementary Materials (overall: Table S1, per bird species - parasite lineage combination: Table S2).

Among avian hosts, five Palearctic migrants, the Garden Warbler (*Sylvia borin*), Icterine Warbler (*Hippolais icterina*), Willow Warbler (*Phylloscopus trochilus*), Spotted Flycatcher (*Muscicapa striata*), and Red-backed Shrike (*Lanius collurio*), and two intra-African migrants, the Woodland Kingfisher (*Halcyon senegalensis*) and Diderick Cuckoo (*Chrysococcyx caprius*), were identified. All remaining identified avian host species were resident. Among migratory species, only three were infected by haemosporidian parasites. The Woodland Kingfisher was infected by a single *Haemoproteus* lineage (HALSEN04) recorded exclusively in this species. The Icterine Warbler harboured a particularly high diversity of *Haemoproteus*, with four distinct lineages (VILWE1, RBQ01, HIICT1, HIICT4), whereas the Spotted Flycatcher was infected with *Haemoproteus* SFC1 and *Leucocytozoon* BT2. The *Haemoproteus* lineage VILWE1 was also detected in the resident Southern Masked Weaver (*Ploceus velatus*), RBQ01 in the Red-billed Quelea (*Quelea quelea*), whereas HIICT1 and HIICT4 were restricted to migrant hosts. All *Plasmodium* and the two other *Leucocytozoon* infections occurred exclusively in resident species. The Red-billed Quelea exhibited the highest parasite diversity, being infected by four *Plasmodium* and nine *Haemoproteus* lineages.

In addition, 1425 mosquitoes were screened for haemosporidian infections, representing 28 species from eight genera (*Aedes*, *Aedeomyia*, *Anopheles*, *Coquillettidia*, *Culex*, *Mansonia*, *Mimomyia*, and *Uranotaenia*). Among them, 76 individuals were infected by 29 *Plasmodium* and two *Haemoproteus* lineages (overall prevalence = 5%, Fig. 2B). Of these, 24 lineages were new, including 23 *Plasmodium* and one *Haemoproteus* lineages. Eight *Plasmodium* and one *Haemoproteus* lineages were highly divergent (>1 % divergence) from known MalAvi sequences. *Plasmodium* lineages were found in *Culex*, *Mansonia*, *Aedes*, and *Coquillettidia* species, while *Haemoproteus* was only detected in *Culex* and *Mansonia*. *C. pipiens* exhibited the highest parasite diversity, being infected by nine *Plasmodium* and one *Haemoproteus* lineages. Mixed infections were detected in eight mosquitoes, six were resolved by manual phasing and two remained undetermined. Infection prevalence for each mosquito species is provided in Supplementary Materials (overall: Table S3, per mosquito species - parasite lineage combination: Table S4).

Compared to the MalAvi database, 34% of avian host-parasite associations (19 of 56) and 7% of mosquito-parasite associations (two of 29, considering only mosquito specimens identified to species level) corresponded to previously known interactions. Most parasite lineages were host-specific, infecting only a single (52 lineages) or two species (nine lineages), with four lineages detected in three species and a further three lineages found in four species. Across both avian hosts and vectors, *Plasmodium* MALNI02 was the only lineage shared between birds and mosquitoes, occurring in *Crithagra flaviventris* and in *Culex* sp., *Mansonia africana*, and *Mansonia uniformis*.

Three lineages previously recorded on other continents, *Plasmodium* AEDVEX01 and ORW1, as well as *Haemoproteus* HIICT4, were identified for the first time in birds in Africa. Similarly, *Plasmodium* CXPER01 (previously reported in Europe) and BT7 (reported from Asia, Europe, and the Americas) were newly recorded in African mosquitoes.

## 4 Discussion

Our study provides an overview of the diversity of haemosporidian parasites in avian hosts and mosquito vectors of southern Africa. We found a remarkably high number of haemosporidian lineages, including 40 in birds and 31 in mosquitoes. Many of these lineages are newly described, which confirms that, despite its large bird and vector diversities, southern Africa remains an under-sampled region. The limited overlap between the lineages detected in birds and mosquitoes suggests that only part of the parasite community was captured, possibly due to local microhabitat variation or seasonal changes in vector activity.

The presence and number of parasite lineages varied substantially among avian orders and families, with some groups exhibiting particularly low infection rates and lineage diversity, consistent with previous studies (e.g., Arriero and Møller, 2008; González et al., 2014; Lutz et al., 2015; Matthews et al., 2016; Perrin et al., 2023). Notably, no haemosporidian parasites were detected in the order Charadriiformes, despite their important presence in the Okavango Delta, an area that is highly favourable for mosquito development (Buxton et al., 2021). This absence of infection is usually explained by a combination of ecological, physiological and biotic factors, such as the scarcity of suitable vectors in certain habitats, host immunological defences, or mismatched host-parasite assemblages (Martínez-Abraín et al., 2004).

The Red-billed Quelea exhibited the highest parasite diversity in our dataset, a pattern consistent with previous findings for this species in Botswana (e.g., Durrant et al., 2007). The high parasite diversity observed in this species has been hypothesised to result from a combination of factors, including its abundance, large population sizes, long-distance movements, and host immune characteristics. However, in the present study, the number of individuals sampled was higher for the Red-billed Quelea than for the other bird species. As a result, while our findings corroborate the high lineage richness previously reported for this species (Durrant et al., 2007), differences in parasite richness among host species should be interpreted with caution, as our dataset may not allow robust comparisons across species.

Several haemosporidian lineages were widespread and cosmopolitan, demonstrating the global connectivity of avian malaria parasites via migratory routes (Perrin et al., 2025). Some of these lineages had previously been reported on other continents, but this is the first time they have been documented in Africa. For example, *Plasmodium* lineage BT7, known from Asia, Europe and the Americas, was found in *C*. *pipiens* in the current study. This finding suggests that there are competent mosquito vectors in Africa that can transmit generalist *Plasmodium* lineages. Similarly, the *Plasmodium* lineage AEDVEX01, which was previously recorded in the migratory bird *Emberiza melanocephala* in Serbia (Stanković et al., 2019), was found here in the resident African species *Pycnonotus tricolor*. This provides direct evidence that parasite lineages infecting European migrants can also circulate among resident birds in Africa. Likewise, the *Haemoproteus* lineage HIICT4, previously reported in *H*. *icterina* in Germany, Sweden and Russia (Reullier et al., 2006; Ellis et al., 2020), was detected in this same species in Botswana. This supports the hypothesis that migratory birds can transport parasites between their breeding and overwintering grounds. Finally, the *Plasmodium* lineage ORW1 was detected in the dark-capped bulbul (*P*. *tricolor*) for the first time in Africa. This lineage is known to infect many bird species, including the long-distance migrant *P*. *trochilus*, which was infected by this lineage in Europe (UK, Bensch et al., 2000). This result demonstrates that a competent vector transmitting this lineage is present in southern Africa and highlights the broad avian host tolerance and ecological plasticity of some haemosporidian lineage.

Migratory birds can carry haemosporidian parasites across continents (Jourdain et al., 2007; Buczek et al., 2020). However, as highlighted by Hellgren et al. (2007), only a few lineages have been confirmed to circulate between breeding and wintering areas, and most haemosporidian transmission appears largely restricted to one geographic region. Interestingly, our results indicate that the migratory species *H*. *icterina* shared two *Haemoproteus* lineages (RBQ01 and VILWE1) with the resident species *P*. *velatus* and *Q*. *quelea*. This suggests parasite compatibility and the potential for cross-transmission across avian host species.

The only parasite found in both birds and vectors was *Plasmodium* MALNI02, which was detected in several species of mosquitoes (*Culex* sp., *M*. *africana* and *M. uniformis*) and a resident bird (*C*. *flaviventris*). This parasite has previously been recorded in resident birds in Malawi (Lutz et al., 2015), Benin (Harvey and Voelker, 2017), Gabon and São Tomé and Príncipe (Loiseau et al., 2017). However, until now, it had never been associated with a vector. Therefore, our detection of MALNI02 in multiple mosquito taxa provides the first evidence of its potential transmission pathway in Africa, although molecular detection alone does not allow confirmation of vector competence and may reflect transient or abortive infections or residual parasite DNA. Nevertheless, the repeated detection of this lineage in several mosquito taxa suggests that *Culex* and *Mansonia* species are likely involved in its local transmission. Overall, these findings suggest that haemosporidian transmission in Botswana is predominantly local, with most lineages circulating among resident birds. While migratory birds may occasionally introduce new lineages or maintain infections acquired elsewhere, they likely act as transient or non-contributing hosts during the non-breeding season.

Most of the haemosporidian lineages detected in mosquitoes were previously unknown, reflecting the current lack of knowledge on African vector-parasite associations. However, several *Plasmodium* lineages (SGS1, SW2, BT7, and GBCAM1) and one *Haemoproteus* lineage (PARUS1) have already been reported from a wide range of avian hosts and a few mosquito vectors, with widespread distributions across Europe and Africa (except for BT7, newly recorded here). Previous molecular surveys conducted in Botswana have reported a total of 36 haemosporidian lineages infecting avian hosts (Waldenström et al., 2002; Beadell et al., 2006; Durrant et al., 2007; Ishtiaq et al., 2012). Of the 40 lineages detected in birds in the present study, 10 correspond to lineages previously recorded in the country, while the remaining lineages represent new records for Botswana. To our knowledge, no avian haemosporidian lineages had previously been described from mosquitoes in Botswana, and all 31 lineages detected in mosquito vectors here are therefore reported for the first time in this context. These findings highlight substantial gaps in our understanding of avian host and vector diversity in the region and underscore the need for broader ecological and parasitological investigations, including studies on parasite dynamics across different habitats and regions (e.g., Namibia and other southern African countries). Expanding the geographic sampling within Botswana and neighbouring areas would also provide a more comprehensive picture of vector-parasite interactions and transmission patterns.

The detection of *Haemoproteus* PARUS1 in mosquitoes is unexpected as this parasite is usually transmitted by biting midges and such findings are rare (Ishtiaq et al., 2008; Njabo et al., 2011). However, DNA detection may result from contamination by traces of a previous blood meal on a vertebrate host infected by *Haemoproteus* lineage, even though all analysed mosquitoes were unfed.

The picture of global transmission between Europe and Africa is complete with the presence of *Plasmodium* SW2 and SGS1. These two lineages have previously been found in resident and migratory birds on both continents. Additionally, *Plasmodium* SW2 has been recorded in *C*. *pipiens* in Europe (Switzerland, Glaizot et al., 2012), and our results demonstrate that this lineage is present in *C. pipiens* in Africa. Similarly, *Plasmodium* SGS1 has been reported in *Culex perexiguus* in Spain (Ferraguti et al., 2013), and our results confirm the involvement of this same vector species in Africa.

Although several of the haemosporidian lineages detected in this study have previously been associated with pathogenic effects in birds, the epidemiological and health implications of the infections reported here remain difficult to assess. For example, *Plasmodium* lineages such as SGS1 and SW2 have been shown to cause disease or measurable fitness costs in some avian hosts (Palinauskas et al., 2008; Ellis et al., 2015; Videvall et al., 2020; Valkiūnas et al., 2024; but see Pigeault et al., 2024). However, pathogenicity is highly host-dependent, and infections by the same parasite lineage can result in markedly different disease outcomes among bird species (Palinauskas et al., 2008). As none of the lineage-host combinations documented here have been experimentally or clinically evaluated, our molecular detections cannot be used to infer infection severity, pathogenicity, or disease risk for the sampled avian species. Consequently, the presence of known lineages should be interpreted as evidence of parasite circulation rather than as an indication of health impact on local bird populations.

In conclusion, our findings provide an integrated picture of haemosporidian parasites present in avian hosts and mosquito vectors in southern Africa, as well as the potential links between migratory bird introductions and local parasite transmission. Several cosmopolitan lineages detected here confirm that migratory birds can contribute to the intercontinental movement of haemosporidians. Once introduced, local vector communities, particularly species of *Culex*, which appear to be important and widespread avian malaria vectors in the region, facilitate their transmission within resident bird populations. This highlights the need to further characterise vector competence across mosquito taxa and to integrate vector and vertebrate host data to better understand parasite circulation across continents.

## CRediT authorship contribution statement

**Antoine Perrin:** Writing – review & editing, Writing – original draft, Visualization, Software, Project administration, Methodology, Investigation, Formal analysis, Data curation. **Casper Nyamukondiwa:** Writing – review & editing, Validation, Supervision, Resources, Investigation, Funding acquisition, Data curation. **Romain Pigeault:** Writing – review & editing, Validation, Resources, Project administration, Investigation, Data curation, Conceptualization. **Mmabaledi Buxton:** Writing – review & editing, Resources, Investigation, Data curation, Conceptualization. **Alexander N. Kirschel:** Writing – review & editing, Resources, Investigation, Data curation. **Molly Baur:** Writing – review & editing, Resources, Data curation. **Olivier Glaizot:** Writing – review & editing, Validation, Supervision, Resources, Project administration, Methodology, Investigation, Data curation, Conceptualization. **Philippe Christe:** Writing – review & editing, Writing – original draft, Validation, Supervision, Resources, Project administration, Methodology, Investigation, Funding acquisition, Data curation, Conceptualization.

## Conflict of interest

The authors declare that they have no known competing financial interests or personal relationships that could have appeared to influence the work reported in this paper.
