# Supplementary material for: Avian haemosporidian diversity and transmission across birds and mosquitoes in Botswana
Source: Int J Parasitol Parasites Wildl. 2026 Feb 14;29:101212. doi: 10.1016/j.ijppaw.2026.101212 (PMC12930081; doi:10.1016/j.ijppaw.2026.101212)
Supplement: Multimedia component 1 [file mmc1.docx]

**Supplementary Method 1:** PCR amplification and sequencing of mitochondrial COI and cytochrome b genes

Uncertain bird species identification was confirmed by PCR amplification and sequencing of the mitochondrial *cytochrome c oxidase subunit I* (COI) and/or *cytochrome b* (cyt b) genes. The COI fragment was amplified using primers LCO1490 and HCO2198, while cyt b was amplified using primers L14841 and H15149. PCR reactions were carried out in a total volume of 25 µl, containing approximately 2µL of template DNA, 1× PCR buffer, 1 mM MgCl₂, 0.2 µM of each dNTP, 0.5 µM of each primer, and 0.6 U of GoTaq G2 DNA polymerase (promega).

Thermal cycling conditions for COI consisted of an initial denaturation at 95 °C for 3 min, followed by 35 cycles of denaturation at 95 °C for 30 s, annealing at 48 °C for 30 s, and extension at 72 °C for 60 s, with a final extension at 72 °C for 7 min. For cyt b, cycling conditions included an initial denaturation at 95 °C for 3 min, followed by 35 cycles of denaturation at 95 °C for 30 s, annealing at 54 °C for 30 s, and extension at 72 °C for 60 s, with a final extension at 72 °C for 7 min.

PCR products were visualised on 1.5% agarose gels. Successful amplicons were purified and sequenced in both directions using the same primers as for amplification.

**Supplementary Table S1.** Number of individuals screened and identified as infected by haemosporidian parasites for each avian host species included in the study.

| bird species | infected | screened |  | bird species | infected | screened |
| --- | --- | --- | --- | --- | --- | --- |
| *Acrocephalus gracilirostris* | 1 | 1 |  | *Mirafra rufocinnamomea* | 0 | 1 |
| *Actophilornis africanus* | 0 | 7 |  | *Muscicapa striata* | 1 | 2 |
| *Anthoscopus minutus* | 0 | 1 |  | *Nilaus afer* | 1 | 1 |
| *Apalis flavida* | 0 | 1 |  | *Oena capensis* | 2 | 2 |
| *Batis molitor* | 1 | 1 |  | *Phylloscopus trochilus* | 0 | 2 |
| *Camaroptera brevicaudata* | 0 | 7 |  | *Ploceus intermedius* | 2 | 3 |
| *Cercotrichas leucophrys* | 0 | 4 |  | *Ploceus velatus* | 9 | 9 |
| *Chlorophoneus sulfureopectus* | 0 | 1 |  | *Ploceus xanthops* | 1 | 1 |
| *Chrysococcyx caprius* | 0 | 1 |  | *Pogoniulus chrysoconus* | 0 | 4 |
| *Cinnyris mariquensis* | 0 | 2 |  | *Prinia subflava* | 0 | 1 |
| *Cinnyris venustus* | 0 | 1 |  | *Pterocles bicinctus* | 0 | 2 |
| *Cisticola chiniana* | 1 | 10 |  | *Pterocles burchelli* | 0 | 2 |
| *Cisticola juncidis* | 0 | 2 |  | *Pycnonotus nigricans* | 1 | 7 |
| *Corythaixoides concolor* | 0 | 1 |  | *Pycnonotus tricolor* | 3 | 12 |
| *Corythornis cristatus* | 0 | 1 |  | *Pytilia melba* | 4 | 9 |
| *Crithagra atrogularis* | 2 | 2 |  | *Quelea quelea* | 47 | 122 |
| *Crithagra flaviventris* | 10 | 10 |  | *Spilopelia senegalensis* | 0 | 1 |
| *Dicrurus adsimilis* | 0 | 2 |  | *Streptopelia capicola* | 13 | 16 |
| *Estrilda erythronotos* | 1 | 13 |  | *Sylvia borin* | 0 | 1 |
| *Euplectes albonotatus* | 0 | 1 |  | *Sylvia subcoerulea* | 0 | 1 |
| *Euplectes axillaris* | 1 | 2 |  | *Trachyphonus vaillantii* | 0 | 1 |
| *Halcyon chelicuti* | 0 | 2 |  | *Treron calvus* | 0 | 1 |
| *Halcyon senegalensis* | 1 | 4 |  | *Tricholaema leucomelas* | 0 | 4 |
| *Hippolais icterina* | 8 | 15 |  | *Turdoides jardineii* | 1 | 1 |
| *Indicator indicator* | 0 | 1 |  | *Turtur chalcospilos* | 1 | 4 |
| *Lagonostica nitidula* | 1 | 1 |  | *Upupa africana* | 0 | 1 |
| *Lagonostica rhodopareia* | 0 | 1 |  | *Uraeginthus angolensis* | 8 | 19 |
| *Lamprotornis chalybaeus* | 1 | 1 |  | *Uraeginthus granatinus* | 0 | 1 |
| *Lamprotornis nitens* | 0 | 1 |  | *Urocolius indicus* | 0 | 22 |
| *Laniarius bicolor* | 1 | 1 |  | *Vanellus armatus* | 0 | 25 |
| *Lanius collurio* | 0 | 2 |  | *Vanellus crassirostris* | 0 | 2 |
| *Lybius torquatus* | 0 | 5 |  | *Vidua regia* | 0 | 2 |
| *Merops pusillus* | 1 | 8 |  |  |  |  |

**Supplementary Table S2.** Vertebrate host-parasite interactions detected in the study. For each avian host-haemosporidian lineage combination, the number of observations is reported, together with the corresponding GenBank accession number of the parasite lineage. Accession numbers beginning with “PX” correspond to parasite lineages newly observed in this study. Individuals with undetermined haemosporidian infections were excluded.

| parasite genus | lineage name | bird species | interaction frequency | GenBank number |
| --- | --- | --- | --- | --- |
| *Haemoproteus* | AFR120 | *Turtur chalcospilos* | 1 | KM056426 |
| *Haemoproteus* | CRIATR01 | *Crithagra atrogularis* | 1 | PX924994 |
| *Haemoproteus* | CRIATR01 | *Estrilda erythronotos* | 1 | PX924994 |
| *Haemoproteus* | HALSEN04 | *Halcyon senegalensis* | 1 | PX924995 |
| *Haemoproteus* | HIICT1 | *Hippolais icterina* | 2 | DQ000321 |
| *Haemoproteus* | HIICT4 | *Hippolais icterina* | 2 | DQ000323 |
| *Haemoproteus* | MEAPI15 | *Merops pusillus* | 1 | OR133319 |
| *Haemoproteus* | NILAFA01 | *Nilaus afer* | 1 | PX924996 |
| *Haemoproteus* | OENCAP01 | *Oena capensis* | 2 | MT888850 |
| *Haemoproteus* | QUEQUE01 | *Quelea quelea* | 1 | PX924997 |
| *Haemoproteus* | QUEQUE02 | *Quelea quelea* | 1 | PX924998 |
| *Haemoproteus* | QUEQUE03 | *Quelea quelea* | 6 | PX924999 |
| *Haemoproteus* | QUEQUE04 | *Quelea quelea* | 4 | PX925000 |
| *Haemoproteus* | QUERY01 | *Quelea quelea* | 4 | EU810750 |
| *Haemoproteus* | RBQ01 | *Hippolais icterina* | 1 | AF495567 |
| *Haemoproteus* | RBQ01 | *Quelea quelea* | 4 | AF495567 |
| *Haemoproteus* | RBQ07 | *Quelea quelea* | 4 | EF117230 |
| *Haemoproteus* | RBQ11 | *Quelea quelea* | 23 | EF117229 |
| *Haemoproteus* | REB4 | *Quelea quelea* | 2 | DQ847197 |
| *Haemoproteus* | SFC1 | *Muscicapa striata* | 1 | DQ060770 |
| *Haemoproteus* | STRCAP01 | *Streptopelia capicola* | 13 | PX925001 |
| *Haemoproteus* | TURPLE01 | *Turdoides jardineii* | 1 | MG018654 |
| *Haemoproteus* | URAANG01 | *Uraeginthus angolensis* | 2 | PX925002 |
| *Haemoproteus* | URAANG02 | *Uraeginthus angolensis* | 6 | PX925003 |
| *Haemoproteus* | VILWE1 | *Hippolais icterina* | 1 | DQ847181 |
| *Haemoproteus* | VILWE1 | *Ploceus velatus* | 1 | DQ847181 |
| *Haemoproteus* | VIMWE1 | *Crithagra flaviventris* | 7 | MT761640 |
| *Haemoproteus* | VIMWE1 | *Ploceus intermedius* | 1 | MT761640 |
| *Haemoproteus* | VIMWE1 | *Ploceus velatus* | 6 | MT761640 |
| *Leucocytozoon* | BT2 | *Muscicapa striata* | 1 | AY393802 |
| *Leucocytozoon* | LANBIC01 | *Laniarius bicolor* | 1 | PX925005 |
| *Leucocytozoon* | TRPIP2 | *Acrocephalus gracilirostris* | 1 | DQ847215 |
| *Plasmodium* | AEDVEX01 | *Pycnonotus tricolor* | 1 | HQ677623 |
| *Plasmodium* | AEMO01 | *Pytilia melba* | 2 | FJ355919 |
| *Plasmodium* | AEMO01 | *Quelea quelea* | 1 | FJ355919 |
| *Plasmodium* | BATMOL01 | *Batis molitor* | 1 | PX925006 |
| *Plasmodium* | COLL7 | *Crithagra flaviventris* | 2 | DQ368376 |
| *Plasmodium* | COLL7 | *Ploceus velatus* | 1 | DQ368376 |
| *Plasmodium* | COLL7 | *Ploceus xanthops* | 1 | DQ368376 |
| *Plasmodium* | COLL7 | *Quelea quelea* | 1 | DQ368376 |
| *Plasmodium* | GRW09 | *Crithagra flaviventris* | 1 | DQ060773 |
| *Plasmodium* | GRW09 | *Lagonostica nitidula* | 1 | DQ060773 |
| *Plasmodium* | GRW09 | *Pycnonotus tricolor* | 1 | DQ060773 |
| *Plasmodium* | GRW09 | *Uraeginthus angolensis* | 1 | DQ060773 |
| *Plasmodium* | LINOLI01 | *Quelea quelea* | 2 | DQ659554 |
| *Plasmodium* | MALNI02 | *Crithagra flaviventris* | 2 | EU810645 |
| *Plasmodium* | ORW1 | *Pycnonotus tricolor* | 1 | AF254963 |
| *Plasmodium* | PLOVEL01 | *Euplectes axillaris* | 1 | DQ659558 |
| *Plasmodium* | RFF1 | *Quelea quelea* | 1 | DQ847264 |
| *Plasmodium* | SYBOR10 | *Cisticola chiniana* | 1 | DQ368390 |
| *Plasmodium* | SYBOR10 | *Lamprotornis chalybaeus* | 1 | DQ368390 |
| *Plasmodium* | SYBOR10 | *Pytilia melba* | 2 | DQ368390 |
| *Plasmodium* | TERUF02 | *Ploceus intermedius* | 1 | EU810618 |
| *Plasmodium* | WW3 | *Crithagra atrogularis* | 1 | AF495577 |
| *Plasmodium* | WW3 | *Crithagra flaviventris* | 1 | AF495577 |
| *Plasmodium* | WW3 | *Ploceus velatus* | 1 | AF495577 |

**Supplementary Table S3.** Number of individuals screened and identified as infected by haemosporidian parasites for each mosquito species included in the study.

| mosquito species | infected | screened |
| --- | --- | --- |
| *Aedes aegypti* | 2 | 2 |
| *Aedes luteocephalus* | 0 | 1 |
| *Aedes mcintoshi* | 0 | 3 |
| *Aedes scatophagoides* | 0 | 1 |
| *Aedes* sp. | 6 | 13 |
| *Aedes unilineatus* | 0 | 3 |
| *Aedoemyia furfurea* | 0 | 11 |
| *Anopheles azevedoi* | 4 | 4 |
| *Anopheles coustani* | 0 | 9 |
| *Anopheles gambiae* | 0 | 66 |
| *Anopheles* sp. | 0 | 8 |
| *Anopheles squamosus* | 0 | 5 |
| *Coquillettidia chrysosoma* | 0 | 65 |
| *Coquillettidia fuscopennata* | 1 | 92 |
| *Coquillettidia metallica* | 0 | 6 |
| *Coquillettidia microannulata* | 0 | 2 |
| Coquillettidia sp. | 3 | 11 |
| *Culex mirificus* | 0 | 1 |
| *Culex naevei* | 4 | 22 |
| *Culex perexiguus* | 4 | 10 |
| *Culex pipiens* | 10 | 81 |
| *Culex poicilipes* | 0 | 37 |
| *Culex quinquefasciatus* | 0 | 23 |
| *Culex rima* | 0 | 22 |
| *Culex simpsoni* | 0 | 1 |
| *Culex* sp. | 8 | 50 |
| *Culex tigripes* | 0 | 1 |
| *Culex univitattus* | 5 | 111 |
| *Mansonia africana* | 12 | 322 |
| *Mansonia uniformis* | 17 | 437 |
| *Mimomyia mimomyiaformis* | 0 | 1 |
| *Mimomyia splendens* | 0 | 2 |
| *Uranotaenia* sp. | 0 | 2 |

**Supplementary Table S4.** Invertebrate host-parasite interactions detected in the study. For each mosquito species-haemosporidian lineage combination, the number of observations is reported, together with the corresponding GenBank accession number of the parasite lineage. Accession numbers beginning with “PX” correspond to parasite lineages newly observed in this study. Individuals with undetermined haemosporidian infections were excluded.

| parasite genus | lineage name | mosquito species | interaction frequency | GenBank number |
| --- | --- | --- | --- | --- |
| *Haemoproteus* | CULPIP03 | *Culex pipiens* | 1 | PX925004 |
| *Haemoproteus* | PARUS1 | *Mansonia africana* | 2 | AF254977 |
| *Haemoproteus* | PARUS1 | *Mansonia uniformis* | 1 | AF254977 |
| *Plasmodium* | AEDSP01 | *Aedes* sp. | 1 | PX960803 |
| *Plasmodium* | AEDSP02 | *Aedes* sp. | 1 | PX960804 |
| *Plasmodium* | AEDSP03 | *Aedes* sp. | 1 | PX960805 |
| *Plasmodium* | BT7 | *Culex pipiens* | 3 | AY393793 |
| *Plasmodium* | CULNAV01 | *Culex naevei* | 1 | PX925007 |
| *Plasmodium* | CULNAV02 | *Culex naevei* | 1 | PX925008 |
| *Plasmodium* | CULNAV03 | *Culex naevei* | 1 | PX925009 |
| *Plasmodium* | CULNAV03 | *Culex univitattus* | 1 | PX925009 |
| *Plasmodium* | CULPER01 | *Culex perexiguus* | 1 | PX925010 |
| *Plasmodium* | CULPER02 | *Culex perexiguus* | 1 | PX925011 |
| *Plasmodium* | CULPIP01 | *Culex pipiens* | 1 | PX925012 |
| *Plasmodium* | CULPIP02 | *Culex pipiens* | 1 | PX925013 |
| *Plasmodium* | CULPIP04 | *Culex pipiens* | 1 | PX925014 |
| *Plasmodium* | CULPIP05 | *Culex pipiens* | 1 | PX925015 |
| *Plasmodium* | CULPIP06 | *Culex pipiens* | 1 | PX925016 |
| *Plasmodium* | CULPIP07 | *Culex pipiens* | 1 | PX925017 |
| *Plasmodium* | CULSP01 | *Culex* sp. | 1 | PX960806 |
| *Plasmodium* | CULSP02 | *Culex* sp. | 1 | PX960807 |
| *Plasmodium* | CULSP03 | *Culex* sp. | 1 | PX960808 |
| *Plasmodium* | CULSP04 | *Culex* sp. | 1 | PX960809 |
| *Plasmodium* | CULUNI01 | *Culex univitattus* | 2 | PX925018 |
| *Plasmodium* | CULUNI02 | *Culex univitattus* | 1 | PX925019 |
| *Plasmodium* | CXPIP36 | *Culex perexiguus* | 1 | PQ798938 |
| *Plasmodium* | CXPIP36 | *Culex univitattus* | 2 | PQ798938 |
| *Plasmodium* | GBCAM1 | *Culex pipiens* | 1 | DQ847267 |
| *Plasmodium* | MALNI02 | *Culex* sp. | 1 | EU810645 |
| *Plasmodium* | MALNI02 | *Mansonia africana* | 1 | EU810645 |
| *Plasmodium* | MALNI02 | *Mansonia uniformis* | 2 | EU810645 |
| *Plasmodium* | MANAFR01 | *Mansonia africana* | 1 | PX925020 |
| *Plasmodium* | MANAFR02 | Coquillettidia sp. | 1 | PX925021 |
| *Plasmodium* | MANAFR02 | *Mansonia africana* | 3 | PX925021 |
| *Plasmodium* | MANUNI01 | *Mansonia uniformis* | 1 | PX925022 |
| *Plasmodium* | SGS1 | *Aedes* sp. | 1 | AF495571 |
| *Plasmodium* | SGS1 | *Coquillettidia fuscopennata* | 1 | AF495571 |
| *Plasmodium* | SGS1 | *Culex perexiguus* | 1 | AF495571 |
| *Plasmodium* | SW2 | *Aedes* sp. | 1 | AF495572 |
| *Plasmodium* | SW2 | *Culex pipiens* | 1 | AF495572 |
